# Current HLA Investigations on SARS-CoV-2 and Perspectives

**DOI:** 10.3389/fgene.2021.774922

**Published:** 2021-11-29

**Authors:** Venceslas Douillard, Erick C. Castelli, Steven J. Mack, Jill A. Hollenbach, Pierre-Antoine Gourraud, Nicolas Vince, Sophie Limou, for the Covid-19|HLA & Immunogenetics Consortium and the SNP-HLA Reference Consortium

**Affiliations:** ^1^ Université de Nantes, CHU Nantes, Inserm, Centre de Recherche en Transplantation et Immunologie, UMR 1064, ITUN, Nantes, France; ^2^ Unesp - Universidade Estadual Paulista, Botucatu-SP, Brazil; ^3^ Division of Allergy, Immunology and Bone Marrow Transplantation, Department of Pediatrics, School of Medicine, University of California, San Francisco, CA, United States; ^4^ Department of Neurology, University of California, San Francisco and Department of Epidemiology and Biostatistics, University of California, San Francisco, CA, United States; ^5^ Ecole Centrale de Nantes, Department of Computer Sciences and Mathematics in Biology, Nantes, France

**Keywords:** MHC, HLA, association analysis, SARS- CoV-2, COVID-19, immunogenetics

## Abstract

The rapid, global spread of the SARS-CoV-2 virus during the current pandemic has triggered numerous efforts in clinical and research settings to better understand the host genetics’ interactions and the severity of COVID-19. Due to the established major role played by MHC/HLA polymorphism in infectious disease course and susceptibility, immunologists and geneticists have teamed up to investigate its contribution to the SARS-CoV-2 infection and COVID-19 progression. A major goal of the Covid-19|HLA & Immunogenetics Consortium is to support and unify these efforts. Here, we present a review of *HLA* immunogenomics studies in the SARS-CoV-2 pandemic and reflect on the role of various HLA data, their limitation and future perspectives.

## The Role of Immunogenetics in Infectious Diseases

### The SARS-CoV-2 Pandemic

In late 2019, hospitals in Wuhan, China, received patients with pneumonia symptoms of unknown origin (Zhu et al., 2020). Researchers quickly identified the cause of this disease, a novel member of the coronavirus family, a single-strand RNA virus further named SARS-CoV-2 by the WHO on February 11th, 2020. This infection lead to COVID-19 disease. It can progress towards the development of an acute respiratory distress syndrome (ARDS) which can be lethal, especially but not exclusively, in older patients and patients with comorbidities (Ruan et al., 2020; Zhou et al., 2020). Previous coronavirus outbreaks, in 2003 with SARS-CoV and 2012 with MERS, had already demonstrated the danger of these known zoonotic viruses for humans (Shi and Hu, 2008). Contrary to SARS-CoV and MERS, which were successfully contained, but caused almost a thousand deaths each, SARS-CoV-2 is still active and endangering human health. In the span of almost 2 years, the virus spread to at least 240 million individuals, leading to more than 4.8 million deaths across the globe (O (2021).oronavir, 2021). The greater scale of this pandemic may be explained by the higher rates of transmission observed, the common asymptomatic carriers and the various severity of infected people (Syangtan et al., 2021).

Researchers determined that SARS-CoV-2 shares 50–79.5% of global sequence similarity with MERS and SARS-CoV, respectively, and that the mechanism of SARS-CoV-2 infection is similar to SARS-CoV, such as highlighted by Guo et al. (Guo et al., 2020). Their viral spike protein, found on the envelope, binds to the ACE2 receptor to enter human cells. While the virus spread globally and on a large scale, multiple SARS-CoV-2 strains have now emerged as the virus mutates, particularly presenting variations in the spike protein, such as the Gamma variant (P.1) in Brazil and the Delta variant (B.1.617.2) in India. These new strains provide a great incentive to assess the possible effects on immunity of such modifications (Burki, 2021), mainly because vaccines were designed to target the original spike protein.

Understanding the host response and the effect of host genomics is key for understanding variation in disease course subsequent to SARS-CoV-2 infection. Initial reports about COVID-19 suggested a pathogenic role of the immune system in the disease, damaging the lungs in a cytokine-storm provoked by CD4^+^ T lymphocytes and monocytes (Zhou et al., 2020). This excessive reaction in the wake of SARS-CoV-2 infection seems to be confirmed in non-human primates with less severe illness in animals with anti-inflammatory responses (Fahlberg et al., 2020). The COVID Human Genetic Effort has investigated these cellular responses at the genetic and genomic levels, describing rare variants in the *IFN* and *TLR* genes in patients with severe symptoms (Zhang et al., 2020a; Bastard et al., 2020; Zhang et al., 2020b; Casanova et al., 2020). Additionally, association studies have identified polymorphisms in the chemokine receptors and IFN, validating their role (Pairo-Castineira et al., 2020; The Severe Covid-19, 2020; D-19 Host Genetics In, 2021). On the genomic level, multiple studies have identified potentially important genes for COVID-19 severity and susceptibility, and researchers organized in different consortia, such as the COVID-19 Host Genetics Initiative, have collected association studies for meta-analyses (Pairo-Castineira et al., 2020; The Severe Covid-19, 2020; Mayoral et al., 2020; Ganna et al., 2020; Castro de Moura et al., 2021). In the same collective spirit, the COVID-19|HLA & Immunogenetics Consortium was created to investigate the role of the most polymorphic region of the human genome, the Major Histocompatibility Complex (MHC), in particular the Human Leukocyte Antigen (HLA) genes which are known to be highly associated with infectious diseases (Chen et al., 2011; Garcia et al., 2013; Spínola, 2016; Sawai et al., 2018; Thoens et al., 2018; Sanchez-Mazas, 2020a). In this review, we acknowledge recent advances linking HLA variation with COVID-19 and advocate for further progress in these efforts.

### Linking HLA and Infectious Diseases: From SNP to HLA Allele

In the past decade, genome-wide association studies (GWAS) have become an essential tool for exploring the link between genetic background and complex phenotypes (Visscher et al., 2017). Rather than focusing efforts on candidate genes, DNA genotyping chips recover Single Nucleotide Polymorphisms (SNP) genotypes along the entire genome. Significant genotype-phenotype associations can be identified by comparing the SNP frequency in one population with a continuous trait (e.g., height, viral load) or between two populations differing by a binary trait or disease (e.g., HIV-1 infected patients vs general population). Contrary to Mendelian genetics, GWAS results are characterized by common genetic variants (allelic frequency ≥0.5–1%) associated with a low to moderate effect size on the outcome of interest, illustrating the “common variant-common disease” hypothesis. Identification of individual SNP contributions allows an overall burden evaluation of the disease genetic risk (Khera et al., 2018) (or protection) and a better understanding of molecular pathophysiological pathways. The GWAS catalog (EMBL-EBI, 2021) was created in 2008 to compile all GWAS results (Welter et al., 2014; MacArthur et al., 2017) and now contain 300,000 associations from 5,000 independent studies (October 6th, 2021).

**FIGURE 1:**
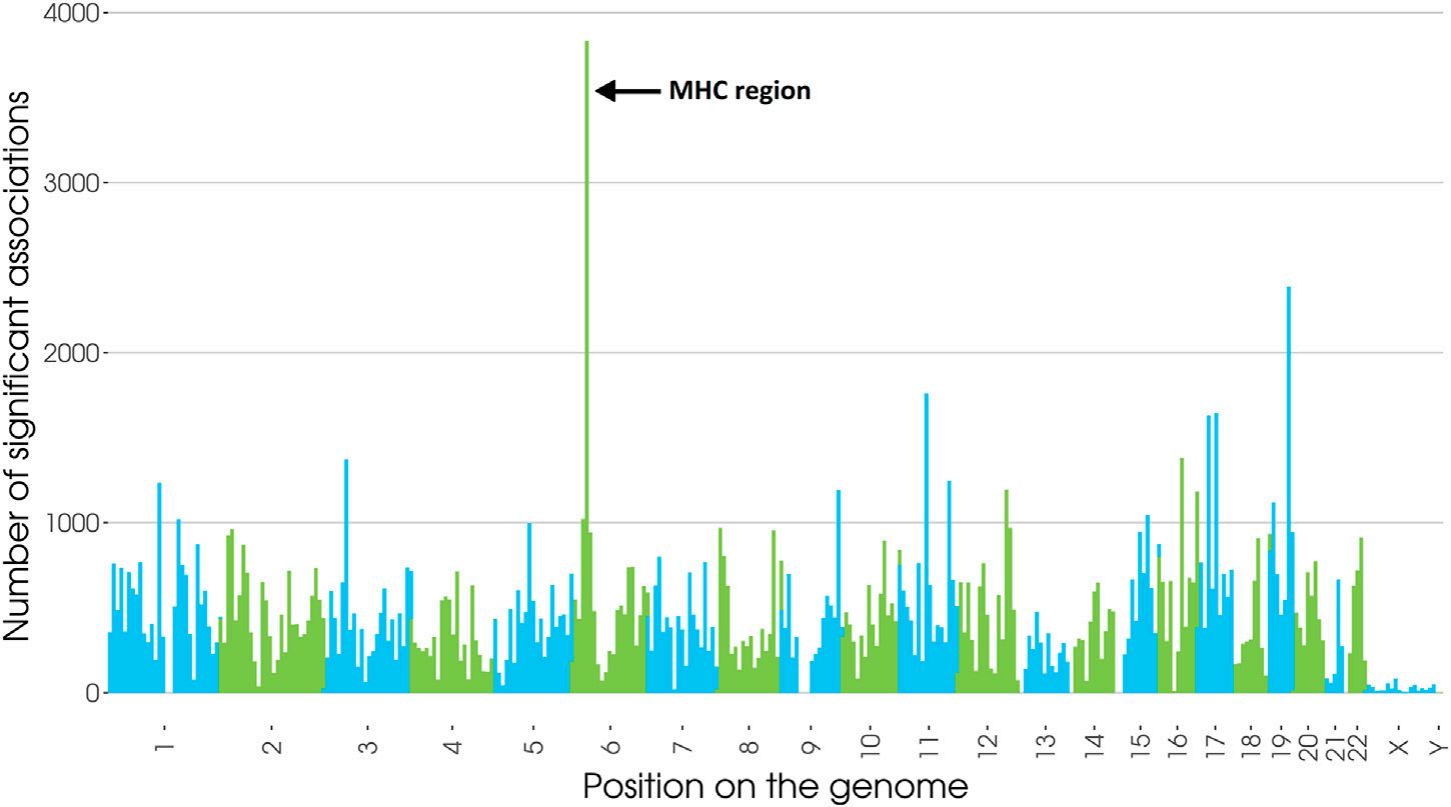
Number of significant SNP associations to any trait or pathology from the GWAS catalog within the whole genome divided in 350 bins (inspired from Lenz TL et al., 2016 (Lenz et al., 2016) & Kennedy et al., 2017 (Kennedy et al., 2017)). 4,080 associations fall in the extended MHC region (25–34Mb, GRCh38), 2,784 of which are located between 30 and 34 Mb where most *HLA* genes are found. Updated on January 14th, 2021.155,456 associations with *p*-value<5 × 10^−8^.

Numerous SNPs in the vicinity of *HLA* genes were confirmed to be associated with diseases (Price et al., 1999), and, the extended MHC accounts for 2.5% of all significant associations (Figure 1), and a third of significant chromosome 6 associations.

Additionally, 21% of all traits in the catalog have at least one association in the extended *MHC*, illustrating the crucial role of *MHC* polymorphisms in human health. As expected, associations near the *MHC* region are immunity-related, from infectious diseases (Sanchez-Mazas, 2020a), to auto-immunity (Dendrou et al., 2018).

For example, one of the most significant *HLA* associations with an infectious disease is for HLA-B*57 tagging SNPs (SNPs not in an *HLA* gene but in linkage disequilibrium, LD, with specific *HLA* alleles) with HIV (OR = 3.47) (Fellay et al., 2007; Limou et al., 2008; Le Clerc et al., 2009; Limou et al., 2009). The rs2395029 SNP, which is almost in complete LD with the HLA-B*57:01 allele, was associated with HIV viral control in Europeans (Limou and Zagury, 2013), and symmetrically, the rs2523608 SNP, likely tagging the HLA-B*57:03 allele was discovered in African-American patients (Pelak et al., 2010). Other viral diseases showed associations with *HLA* SNPs include Hepatitis B virus (Hu et al., 2013; Jiang et al., 2015), Hepatitis C virus (Lee et al., 2018; Vergara et al., 2019), Epstein-Barr virus (Rubicz et al., 2013), and SARS-CoV (Sanchez-Mazas, 2020b). For a review, please see the extensive report by Sanchez and others (Sanchez-Mazas, 2020a).

However, the fact that GWAS identify a large genetic region associated with an outcome, without directly pinpointing functional, causal variants represents an important challenge for interpreting GWAS results. Such interpretation is made even more difficult by the complex LD patterns of the extended *MHC* region. Additional *HLA* typing and statistical inference of *HLA* alleles can refine the SNP association signals to specific *HLA* alleles, reflecting specific molecular functions and pathways. Such a strategy was successfully implemented for infectious diseases such as HIV, HPV, Dengue, and Ebola (Lin et al., 2003; Wang et al., 2011; Nishida et al., 2016; Adebamowo and Adeyemo, 2019; Chen et al., 2019; Ekenberg et al., 2019; Butler-Laporte et al., 2020; Chaisri et al., 2020; Huang et al., 2020; Ursu et al., 2020; Yengo et al., 2020).

### Scope of the Review

Despite the central roles played by the *MHC* region and HLA molecules for the study of immune-related disease, understanding the underlying mechanisms of susceptibility and protection is far from complete (Trowsdale and Knight, 2013). The current pandemic raises questions regarding the role of HLA in recognition of or immune responses to a new virus. In this report, we review the first HLA-related investigations of SARS-CoV-2 and advocate for further efforts in HLA and COVID-19 analyses, using modern algorithms and resources, in order to enhance present and future research.

## COVID-19 and HLA Association Studies

*HLA* polymorphisms have previously been closely associated with viral infections and disease outcomes, whether they are associated with protection or susceptibility. The intrinsic diversity of HLA molecules and the many possibilities to investigate their link to diseases sparked researchers’ interests during this novel pandemic. Researchers have investigated the interaction of host HLA diversity on both the infection by SARS-CoV-2 and the severity of the resulting COVID-19.

### *In Silico* Peptide Binding and HLA Allele Frequencies

Studies using *in silico* peptide binding and *HLA* allele frequencies rely on available databases which do not require to generate data; thus, they are the first actionable steps to HLA analysis. Nguyen et al. proposed the first *in silico* HLA approaches in early 2020 by using the reference amino acid sequence of the SARS-CoV-2 (with NCBI accession number, NCBI:txid2697049) along with the netMHCpan software to predict the class I HLA alleles most susceptible to presenting SARS-CoV-2 peptides (Nguyen et al., 2020). They identified HLA-B*46:01 as the least presenting allele and HLA-B*15:03 as the most presenting one, possible risk and protective factors of infection, respectively. This publication was highlighted in the immunogenetics section of Nature, creating a starting point for HLA researchers (Zahn, 2020). Later, La Porta and others used Artificial Neural Networks to predict the binding capacity of each HLA class I allele, also demonstrating B*46:01 and others as a weak binder, and B*15:03 as a strong binder (La Porta and Zapperi, 2020). However, their results do not entirely overlap, demonstrating that functional studies should be performed. Barquera and others performed a similar analysis also considering *HLA-DRB1* and *HLA-DQA1/DQB1*, indicating many HLA alleles (some highly frequent) among the best presenters, including B*15:03, and another list of worse presenters, including B*46:01 (Barquera et al., 2020).

Interestingly, B*15:03 frequency varies across the globe, with high frequencies in African populations and admixed ones (such as Brazilians), but low frequencies in Asia and Europe. Conversely, B*46:01 is highly frequent in Asia and rare in the rest of the world. The same dynamics can be observed for most of the alleles in the strong or weak presenter list.

Romero-López et al. expanded this investigation to class II HLA alleles and identified multiple HLA-DP and HLA-DR HLA alleles as well as HLA-A*02:03 as the allele with the most binding affinity to a viral peptide (Romero-López et al., 2020). Further research by de Sousa et al. of the most frequent HLA alleles of people from Europe, Asia and Africa and their interaction with variants and seems to point towards a selective pressure of class II MHC only regarding the binding of the ORF8 protein in SARS-CoV-2 (de Sousa et al., 2020).

The first studies only displayed correlations between COVID-19 phenotypes (e.g., incidence, severity, mortality) and *HLA* allele frequencies obtained in the allelefrequencies.net database or from local bone marrow donor registries, notably in Italy, an important European cluster. Correale et al. investigated class I correlations at one-field resolution (Correale et al., 2020). Pisanti et al. took a closer look at HLA haplotypes with an Italian registry, and identified HLA-A*01:01 g ∼ B*08:01g ∼ C*07:01g ∼ DRB1*03:01g as positively correlated with incidence and HLA-A*02:01g ∼ B*18:01g ∼ C*07:01g ∼ DRB1*11:04g as negatively correlated with incidence (Pisanti et al., 2020). Some studies took a more global approach by comparing the COVID-19 statistics of every country to their known *HLA* allele frequencies, providing discordant and mostly non-significant results (Ishii, 2020; Sakuraba et al., 2020; Tomita et al., 2020; Toyoshima et al., 2020). Other studies focused on a cellular level and identified a preponderance of monocytes with low expression of HLA-DR in infection and severity of SARS-CoV-2 (Benlyamani et al., 2020; Zmijewski and Pittet, 2020; Kreutmair et al., 2021; Roussel et al., 2021).

### *HLA* Association Studies

Later, *HLA* association studies of various sample sizes tried to evaluate the direct link between HLA and different COVID-19 phenotypes. Wang et al. inferred the HLA class I and class II genotypes of 332 Chinese individuals to compare severe and mild cases of COVID-19, using xHLA (Xie et al., 2017) and SOAP-HLA (Cao et al., 2013), two software which allow HLA genotyping from sequencing data. HLA-A*11:01 (*p*-value = 0.009, OR 2.3), HLA-B*51:01 (*p*-value = 0.007, OR 3.3), and HLA-C*14:02 (*p*-value = 0.003, OR 4.7) were identified as top signals in the *HLA* class I region (Wang et al., 2020a). Direct *HLA* typing cohorts were also investigated across the world, but with small sample sizes going as high as 190 individuals. No associations were found by Iturrieta-Zuazo et al. in 45 Spanish patients between COVID-19 severity and *HLA* supertypes (Iturrieta-Zuazo et al., 2020), and none was found between mortality at 30 days and *HLA* one-field genotypes from 72 individuals from Canary Islands by Lorente et al. (2021). Three different groups conducted association analyses against a healthy control group to identify susceptibility of infection to SARS-CoV-2: Wang et al. (2020b) compared 82 COVID-19 vs 3,548 controls from China and found HLA-B*15:27 as associated (*p*-value = 0.001, OR 3.6), Novelli et al. (2020) compared 99 COVID-19 vs 1,017 controls from Italy and found 3 significant association (HLA-B*27:07, *p*-value = 0.00001; HLA-DRB1*15:01, *p*-value = 0.002; HLA-DQB1*06:02, *p*-value = 0.0001), and Yung et al. (2020) compared 190 COVID-19 vs 3892. controls from Hong-Kong but did not identify any significant association. More recently, Khor et al. (2021) also identified HLA-A*11:01:01:01 as a risk factor for COVID-19 severity (*p*-value = 0.003, OR 3.4), in a study involving 190 patients and 423 controls, after controlling for comorbidities and other confounding factors. Shachar et al. (2021)showed no association between COVID-19 severity and *HLA* alleles in a large-scale study of *HLA* typed Israelis (n = 20,937), though it was limited to two-field information. Finally, Castro de Moura et al. investigated the relationship between the epigenome of patients and COVID-19 severity from 407 patients and showed differentially methylated probes in *HLA-C* associated with the response of interferon in the viral response (Castro de Moura et al., 2021).

In addition to these studies, the Severe COVID-19 Consortium conducted a genome-wide association study of 1,980 patients of European ancestry and notably investigated *HLA* with classical SNP association, and *HLA* association by NGS genotyping in a subset of individuals. This was the first high-scale genomics initiative. However, chromosome 3 (*SLC6A20*, *LZTFL1*, *CCR9*, *FYC O 1*, *CXCR6*, and *XCR1*) as well as in the ABO locus (with A as risk and O protective) were the only significantly associated loci (The Severe Covid-19, 2020). The absence of *HLA* association was also shown by the meta-analysis on COVID-19 severity performed by the COVID-19 Human Genetic Initiative (HGI), where a variant in *HLA-G* was found but not replicated (Pairo-Castineira et al., 2020). However, the HGI release 6 in June 2021 identified 5 variants reaching statistical significance within the *CCHCR1* gene, situated 110 kb downstream of *HLA-C* (top SNP: rs111837807, *p*-value = 2.2 × 10^−11^, OR_meta_ 1.23) as well as a variant within *HLA-DPB1* 3′UTR (rs9501257, *p*-value = 4.1 × 10^−8^, OR_meta_ 1.19), when comparing the general population to patients with critical COVID-19 (n_cases_ = 8,779, n_control_ = 1,001,875, from 25 studies of various ancestries). It is notable that multiple variants linked to *HLA* genes seemed consistent, but not significant, between studies (D-19 Host Genetics In, 2021), which suggests that increasing cohort sizes in the future or running in-depth HLA-centric explorations may reveal additional significant signals.

## Conclusion

Classical large GWAS meta-analysis recently reported SNP associations in the *MHC* region, mostly with critical COVID-19 illness, however the impact of HLA molecules might not be as imagined for this novel infectious disease. Unlike HIV-1 infection where HLA is the driving signal of viral control and disease progression, impact of HLA in SARS-CoV-2 infection seems milder and mostly restricted to severity symptoms, and its role has yet to be fully understood.

Multiple HLA-focused analyses performed during the last 2 years have had greatly varying results with inconsistent associations even in large studies [n = 20,937 in (Shachar et al., 2021)]. Further direct *HLA* allele association studies could provide the necessary power to carefully assess the role of HLA in immune response against SARS-CoV-2, but unfortunately, typing has not been conducted on large samples to date, leading to underpowered studies (most studies with less than 190 individuals). Indeed, HLA exploration requires large sample size; the HLA system has an important diversity, with thousands of alleles on multiple different genes. In a given population, a few numbers of these alleles are usually sufficient to represent the majority of individuals. However, to understand the role of the HLA system in diseases, it is important to also study alleles with a smaller frequency, which may be absent of cohorts with limited sample size.

*HLA* allele inference from sequencing (WGS and WES) and SNP genotyping data already generated for genome-wide analyses with the support of large biobanks and international consortia should therefore be given a high priority in the near future to provide a definitive answer on the impact of HLA molecules on COVID-19 phenotypes. Indeed, promising results from large association meta-analyses showed associations of both class I and class II *HLA* SNPs with severity, in the latest data release of the COVID-19 Host Genetic Initiative. Furthermore, the study of *HLA* 5-gene haplotype organization, and other immunogenetic parameters such as cell surface expression levels and interaction with KIR ligands may paint a bigger picture on the underlying immunogenetic mechanisms involved in the infection course.

HLA studies reported in this review rely on correlations and moderate size cohorts as stated. However, the COVID-19 crisis created an international collaboration to share data in order to explore host genetics risk factors for different COVID-19 outcomes (D-19 Host Genetics In, 2021). A vast amount of NGS and GWAS data have been generated: 49,562 COVID-19 positive cases vs >2M population controls with GWAS data in the COVID-19 Host Genetics Initiative (D-19 Host Genetics In, 2021); 20,952 cases vs 565,205 controls with WES data in the Regeneron study (Kosmicki et al., 2021). Thinking beyond COVID-19, the large national and international human genomics efforts represent a unique opportunity to promote large-scale HLA-centric analyses and to better describe *HLA* allele diversity across the globe by leveraging novel inference algorithms. These algorithms allow HLA typing from NGS and GWAS data (i.e., xHLA (Xie et al., 2017) and HIBAG (Zheng et al., 2014), respectively). Concerning other immunogenetics parameters, such as 5-gene HLA haplotypes or KIR ligands, it is now possible to infer them with HLA data (Geffard et al., 2020), with a detailed review of these tools in Douillard et al. (2021). Using these tools at a large scale on existing cohorts with GWAS and NGS data will clarify the role of HLA in COVID-19 outcomes and help understanding the mechanisms of the pathology.

The SARS-CoV-2 pandemic has had a huge global health toll, and has sparked a collective effort in the scientific community to identify candidate targets accounting for the diversity in response to the infection. HLA was quickly investigated for links with the SARS-CoV-2 infection and the resulting COVID-19 disease. The first studies, often underpowered, showed discordant results, and more robust association studies recently suggested a much milder effect of *HLA* SNPs and alleles on COVID-19 phenotypes as foreseen. The choice of the phenotype of interest was also proven to be crucial in association studies, as COVID-19 severity seems to be more closely linked to *HLA*. In this report, the COVID-19|HLA & Immunogenetics Consortium aimed to provide a critical view of current *HLA* analyses and their intrinsic power and limitations. We also hope this report will incite geneticists to run HLA-centric studies by expanding the pool of data available for *HLA* genotyping and genotypes imputation, in order to untangle the precise role of the Major Histocompatibility Complex in COVID-19 outcomes and other immune-related diseases.
